# On Plastic Operations

**Published:** 1860-04

**Authors:** B. Langenbeck

**Affiliations:** Professor of Surgery in the University of Berlin


### NEW YORK MONTHLY REVIEW

AND

### BUFFALO MEDICAL JOURNAL.

**VOL. 15.** APRIL, 1860. **NO. 11.**

### ORIGINAL COMMUNICATIONS.

*Osteo-Plastic Operations.* By B. Langenbeck, Professor of Surgery in the University of Berlin. Translated and communicated by William F. Holcomb, M.D.

*Osteo-Plastic* operations are those which have for their purpose the reparation of defects in bone, or the performing of resection in such a manner that the removed portion may be supplied by a *new deposit* of osseous matter.

The reparation of defects in bone may be effected by various operative methods.

I. By bringing together and uniting the borders or edges of the fissures in bone.

The closure of fissures in the hard palate is attempted in this manner.

II. By transplanting a neighboring portion of bone to supply the defect.

The moving and *engrafting* of the ossa intermaxillaria into the fissure of the processus alveolaris, in double-cleft palate, as accomplished according to Blandin's method, by *cutting through the vomer;* Gensoul's plan of breaking up the union of the intermaxillary bones with the vomer, and my operation of dividing the *cartilago-triangularis,* illustrate this method. Pirogoff's proposed plan of transplanting the calcaneus to the sawed surface of the tibia, after a previous exarticulation of the foot and the consolidation of both these bones, belongs also to this class.

III.—The reunion of a fragment of the detached bone.

The first attempts of this kind were made by Percy, who endeavored to supply defects, occasioned by gun-shot wounds in the lower extremities, by engrafting portions of the tibia of an ox. These experiments failed. Von Walther was more fortunate in replacing a piece of cranial bone, trepanned from a man 36 years of age, as the greatest portion of the bony disk healed, while only a small portion exfoliated. The attempt made later by B. Heine, to reunite an exsected portion of the rib of a dog, terminated just as unfortunately as the experiments of Percy; and there is reason to doubt the correctness of Von Walther's observations, since portions of bone, which are *set into* or engrafted into others, suffer *erosion* from long contact with the normal fluids from the bone or from pus, very similar to the destruction in caries or exfoliation. In many cases where I have used ivory pins for the purpose of uniting pseudo-arthroses, after three weeks' use, even these were found to have sustained a considerable loss of substance. The end which had been in the bone was rough, as if eroded by caries, and had lost even a third of its circumference. In the case related by Von Walther, the portion of bone which was removed by the trepan, was replaced and allowed to remain three months, may have become reduced in size by a similar process. This subject has lately acquired a new, though only a physiological interest, through the researches of Ollier. (See the work "On the Artificial Production of Bone by Means of Transplantation of the Periosteum, and by Osseous Grafts. By L. Ollier. 1859.) This author (*v.* page 13) engrafted the bones of animals upon those of the same species, (rabbits,) sometimes under the skin of the axilla, and sometimes in cavities formed by the extirpation of a corresponding bone, (os metatarsi;) the transplanted bones continued to live and grow in their new home. On account of the greater vulnerability of the human species, and its slight recuperative power, as well as the impossibility of employing corresponding material, it is impossible to make these experiments in operative surgery. But that it is possible, however, to effect reunion in a bone which has been completely separated from its *bony* connections, provided it remains united to the parent bone by the periosteum, is shown by the following case:

Case.—*Naso-pharyngeal Polypi; Resection of the Processus Nasalis and the Right Os Nasalis; Extirpation of the Polypi; Replacing and Reunion of the resected Bones.*

A healthy boy, of 18 years, was received into the clinic, in whom the space behind the soft palate was completely filled by two fibrous polypi. The smaller tumor was attached to the vicinity of the *spina nasalis posterior*; and the larger, near the right tuba eustachii; and from it, a prolongation extended into the right nasal cavity, by which it was completely closed. Respiration was difficult, and considerable hæmorrhage had taken place. As it appeared impossible to remove the tumors either through the nasal cavity, or by division of the soft palate and resection of the palate bones through the mouth, I decided to resect the processus nasalis of the superior maxilla, as I had done before in similar cases. (*Vide* Theo. Billroth on Resection of the "Processus Nasalis." Deutsche Klinik. 1854.) In the former cases I had only resected and removed the processus nasalis of the superior maxilla. But as, in this case, the bone referred to was not driven forward, I had reason to fear the passage to the pharynx made by its removal would not be wide enough to enable me to reach the tumor. As the *permanent* resection of the right nasal bone would leave a proportionate disfiguration, I decided on trying to replace the resected bone. The operation was performed Nov. 3d, 1859. A nearly straight incision was made from the *glabella*, passing downward to the right over the processus nasalis, running to the ala nasi. The *skin*, carefully dissected, was so far separated that the processus nasalis of the superior maxilla, and the whole of the right os nasale, were brought into view, and then the upper border of the right ala nasi was detached from its corresponding connections with the above-mentioned bones. By means of sharp bone forceps, an incision was made from the nasal cartilage along the sutura nasalis to its union with the os frontis; and by a second incision, extending into the sinus maxillaris, the base of the processus nasalis was divided. This incision terminated where the angle of the superior maxilla, below the orbit, joins the os lachrymale. An elevator, introduced into the nasal cavity, was used to raise both bones from their bed, and at the same time the nasal bone separated at its suture with the os frontis, so that the whole, adhering to the periosteum, could be thrown up and held on the forehead. The polypi were then removed. After the bleeding was checked, the BONY FLAP was brought to its former place, and the external wound was closed by silver sutures, (which were *tied* like a thread,) and the nasal cavity carefully plugged with *charpie*. These resected bones were completely detached from the superior maxilla, frontal and left nasal bones, but were held together by periosteum and mucous membrane, and were also joined to the ossa frontis and nasal cavity by a strip, about an inch wide, of periosteum and mucous membrane. For a few days after the operation, there was considerable swelling of the soft parts covering the wounded bones, which had, however, nearly disappeared on the 18th of November, by the application of cold water compresses. Cicatrization of the soft parts complete; no secretion from the nasal cavity; and the mucous membrane, as far as can be felt by the finger, appears to be healed. Respiration by the nose is entirely free. Pressure of the finger on the resected bones causes no pain. Between the nasal process and the superior maxilla there exists a little tumefaction, but none at the nasal suture. Patient left the bed.

IV.—"PRESERVATION OF THE PERIOSTEUM AND THE SURROUNDING SOFT PARTS."

All surgeons who have resected bones, acknowledge that, in order to have the bone reproduced, it is of the highest importance that the surrounding periosteum be preserved; and all experience demonstrates that the removed portion of bone will be more or less perfectly reproduced in proportion as the periosteum is preserved. As long ago as 1843, I extirpated the entire *ulna*, together with the carious superior extremity, which was much hypertrophied from a chronic traumatic inflammation of long standing. In this case, the *entire* periosteum was preserved. A new ulna was deposited, with its perfectly traceable processes. The movement of the elbow-joint was complete, and the new, but more flattened, olecranon could be felt through the soft parts. In the year 1846, I brought this young man before the meeting of the German Scientific Congress. (See Official Report of the 24th Congress of German Naturalists and Physicians, in Kiel, 1846.) Since that period, a very great number of resections and extirpations of bone have convinced me that, by preserving the periosteum, a perfect reformation, (osteo-genesis,) or reproduction of bone, may be expected with certainty, unless the bone is suffering from *discrasia*. This opinion, formed before 1848, explains why, in the Schleswig-Holstein war, I undertook the resection of the fragments of broken *diaphyses* of bones, fractured by gun-shot wounds—an operation for which I have been greatly blamed, but which, I am thoroughly convinced, will one day take its place in military surgery. In the resections which occur in times of peace, it is very easy to preserve the periosteum which has become thickened, (as in inflammed bones;) impossible, however, when we have to expect long bones which are attacked by tumors. The preservation of the periosteum, in the resection of joints, is inadmissible, (so far as it *can* be preserved,) in case we wish to obtain a movable joint. These operations, according to our former ideas, offer no inducements for us to try to preserve the normal, *thin* periosteum.

Every operation which *maims* the patient is a "*testimonium paupertatis*" for the surgeon.

This reflection always forced itself upon me at each one of the numerous resections of the upper jaw which I have performed, and, nevertheless, (I am ashamed to confess it,) the idea never occurred to me that it might be possible to bring this operation directly into the field of conservative surgery, by detaching the periosteum from the exsected bones and leaving it in connection with the adhering soft parts. The complete extirpation of the superior maxilla, or of half of it, not only deforms the face in a sad manner, but leaves behind a far worse result as regards articulation and deglutition, because the partition between the mouth and nasal cavity falls away. The conservation of this partition I consider as *certainly secured*, if we do not remove the *involucrum palati duri*, but preserve it, detaching it with the periosteum from the hard palate. I even regard it as possible to preserve the periosteum of the facial and orbital surfaces of the superior maxilla, and thus, perhaps, render the disfigurement less noticeable. It is unnecessary to say, however, that when extirpation is performed on account of *cancer*, this is impossible. But this operation is practicable in the majority of *osteo-sarcomatous*, *fibrous*, *enchondromatous* and *myeloid* growths of the superior maxillary. The detaching of the periosteum from the superior maxillary bones, the surface of which is so irregular, is difficult and troublesome, and thus additional labor in extirpation will detract considerably from the precision and rapidity of the operation. Further experience will show which incision (through the soft parts) will render the detaching of the periosteum the most convenient. In any case, I would commence the most difficult part of the operation by first making an incision along the lower and outer margin of the gums, and then, by means of a *denuding* instrument, would detach the gum with the periosteum from the bone; then divide the gums along the inner surface of the alveolar process as far as the soft palate, and separate it, *with the periosteum*, from the roof of the mouth. Then the incision of the external soft parts, which should be separated from the bone with the periosteum, should follow. The periosteum of the orbit should also be most carefully preserved; and lastly, the *bones* can be divided in the usual manner. I would finally join the gums and the periosteum of the facial surface to the border of the detached *involucrum palati duri* by means of sutures. The cases in which I have operated in this manner are yet too recent to admit of an opinion as regards the reproduction of bone; nevertheless, I may be allowed to communicate them.

FIRST CASE.—*A large Enchondroma on the under surface of the Palatum Durum—Removal of the Tumor—Preservation of the Bone and the Involucrum Palati Duri—Healed by first Intention.*

W., 27 years of age, of healthy appearance, and, excepting having contracted a primary syphilitic affection five years ago, said he was never sick; in 1857, observed a swelling, of the size of a pea, in the left half of the hard palate, which caused no pain. At first, this increased very slowly; but after a while, and particularly since the fall of that year, it grew very rapidly, softened in several places, and ulcerated near the teeth of the left side. The tumor occupied no longer a small surface, but spread over the entire palatum durum, and pressed everywhere upon the fine, sound teeth of the superior maxilla, and when the jaw was closed it nearly filled up the mouth; it also pushed the soft palate posteriorly towards the basilar portion of the os occipitis. The swelling, which was firm, hard, uneven, and free from pain, was covered by the *involucrum palati duri*, which appeared healthy until near the margin of the alveoli of the left side, where the ulceration existed, through which the probe could be introduced into the substance of the tumor. The probe, and also the acupuncture needle, when passed into the swelling as far as possible, encountered everywhere osseous matter scattered throughout the semi-solid mass.

According to the patient, many small pieces of bone had been thrown off. Whether these belonged to the *palatum durum*, or whether they were ossified portions of the tumor, which had been regarded as enchondromatous, could not be decided with certainty. The entire facial surface of the superior maxilla appeared unchanged, except a small bony tumor the size of a cherry, which was situated on the outside of the alveolar processes above the first molar of the left side. The voice was gone; the articulation indistinct; the respiration was difficult when the mouth was closed; the nasal passages were, nevertheless, perfectly free. On examination with the finger, per nasum, the upper surface of the *palati duri* was found to be smooth, and of normal resistance. But in the left nostril, the *inferior concha* felt rough, and it appeared as if there was a communication between the tumor and the bottom of the nasal cavity; as formerly, by pressure on the tumor in the mouth, blood flowed from the left nostril.

According to the first impression which the disease made upon many physicians, (and on myself also,) who examined the patient, we were led to regard the resection of the left half of the *superior maxilla*, and the entire *palatum durum*, as unavoidable; and the young man had decided to have the operation performed in the Clinic, but the sound and firm teeth, as well as the normal condition of the nasal cavity, warranted (after repeated examinations) the hope that such disfiguration might be avoided. The operation was performed Nov. 14, 1859. A firm incision along the inner margin of the teeth of the left superior maxilla divided the *involucrum palati duri*, together with the *periosteum*, which were now loosened partly by means of the raspatory, and partly with the knife.

This part of the operation was very difficult in the vicinity of the ulcerated opening, but became easier in proportion as I advanced posteriorly, and succeeded so completely that both membranes terminating in the soft palate hung down like a flap of skin, and the entire swelling was brought into view. The periosteal surface of this flap was smooth throughout, excepting only in the vicinity of the ulceration, where some fragments of the tumor remained, which were carefully removed. The tumor itself was now (by means of a sharp chisel) separated from the *palatum durum*. Some isolated inequalities of the bony palate (which appeared entirely free from disease) were then removed, and I replaced the flap directly against the bony surface, and fastened it to the gums by a firm suture. A moistened sponge was inserted into the mouth, and held in place against the palate of the tongue. The tumor appeared to be purely enchondromatous, with an abundance of bony scales scattered through it. The whole wound healed by first intention, without the occurrence of the least accident. The flap adhered firmly and smoothly to the bony palate, except at two points in the centre, where it had not united to the bone, without, however, any suppuration having occurred. If a superficial exfoliation should follow this operation, requiring removal, we have the proof that it is possible, even in the resection of the superior maxilla, to preserve the periosteum and *involucrum palati*, and reunite them to the gums, as was done in this case.

SECOND CASE.—*Exostosis of the Processus Alveolaris—Resection of the same, with the preservation of the Gums, Involucrum Palati Duri, and Periosteum.*

H. W., 7½ years old, a boy of very anæmic appearance, was brought to be operated on in the Clinic Nov. 14th, 1859. The enlargement of the *processus alveolaris*, of three years' growth, extended from the gap occasioned by the removal of the upper posterior molar of the right side, to the second incisor of the left side, and extended outward towards the right *ala nasi*. The tumor was about the size of an English walnut, and projected equally inward (towards the hard palate) and outward, so that the upper lip and ala nasi appeared slightly pushed forward. The tumor is hard as bone, painless, except on firm *pressure*, when the pain is so great as to prevent a faithful examination. It was diagnosticated as hypertrophy of the medullary substance of the bone.

Operation.—An incision was made through the gum, near the *processus alveolaris*, from the superior posterior right molar to the second superior incisor of the left side, which had been previously extracted. A similar incision was made on the inner margin of the *processus alveolaris*, through the *involucrum palati duri.* The integuments of the tumor, together with the periosteum, were detached without serious difficulty, by means of a raspatory. The bone was then cut out with bone nippers, in a triangular form, the base looking downward. The second incision crushed the thin plates of the alveolar processes. At this juncture a small, smooth bony substance, about the size and form of a musket-ball, sprang out of the opening. The soft coverings of the alveolar processes were now brought into place, and the edges of the wound exactly joined by a suture. During the first days following the operation only a slight swelling was observable, and the immediate healing of the wound seemed certain. On the fourth day, while the patient was feeling perfectly well, there appeared (in consequence, probably, of some mental agitation,) an unimportant arterial hæmorrhage from the wound. The bleeding ceased after removing the sutures, and syringing the wound with cold water. It returned, however, the following day, and was arrested by the application of a tampon of tannin. On November 21st it reappeared; small pellets of lint, wet in tinct. ferri sesquichloridi, were pushed into the cavity, and effectually checked the hæmorrhage. Previous to the first bleeding, the edges of the gums and of the involucrum palati duri came so closely together that the defect in the bone was completely covered; only the teeth appeared to be wanting. Notwithstanding the union was destroyed by the hæmorrhage, yet I hope to be able to repair the defect in the bone at some future period. Since non-malignant tumors of the alveolar process of the superior and inferior maxillary bones very often occur, and as, until now, the soft parts covering them have always been removed *with the tumors*, therefore I regard this sub-periosteal resection, which may be resorted to in the majority of these cases, as a real progress in this operation.

On November 15th, 1859, this case prompted me to try to detach the periosteum, and also the *involucrum palati duri*, in the total extirpation of the right superior maxilla of a boy, 14 years of age. The attempt failed, the mouth and pharynx being nearly filled by this fibrous tumor of the superior maxilla, which had been previously, and for a long time, treated by caustics, in the hope of destroying it. This cauterization was carried on with great energy, and by means of some caustic unknown to me. At the time the boy was received into the Clinic, on that part of the tumor which protruded from beneath the upper lip there was still a suppurating surface, and the *involucrum palati duri* appeared to be degenerated into an indurated, granulating mass. The soft parts immediately covering the *superior maxilla* had become so friable, in consequence of the long-continued suppuration, that they crumbled into pieces as I attempted to detach them from the bones. The covering of the *palatum durum* and the gums, together with the periosteum, should have been *detached* from the bones, but I only succeeded in *detaching* a large portion which covered the facial surface of the bone. As regards the reproduction of the bone in this case, I shall report at a future period.

V.—Transplantation of the Periosteum upon Defects in Bone.

The experiments of Ollier upon animals (rabbits) have greatly enlightened us in regard to the *power of the periosteum in the reproduction of bone.* He separated flaps of periosteum from the tibia, in such a manner that they remained attached to the bone only by a small strip, and inserted the free end between the muscles of the limb. After a comparatively short time, an *exostosis* had sprung from the flap thus transplanted, the growth of which did not cease, even when, a few days after the transplantation, the strip connecting it to the parent bone was divided. So active was the *independent power* in the periosteum of *forming bone*, that when flaps of it were entirely detached from the tibia, and inserted into the axilla of the same animal, or of another of the same species, it continued to produce bone. To what extent these observations may be applicable in operative surgery experience must determine. *A priori*, we should expect that *human periosteum* will perform its functions as well, when it can be transplanted under as favorable circumstances, as in the cases of Ollier; that is, if we can convey to the living tissues detached periosteum, before it has lost its temperature and natural vitality, and insure immediate reunion of the wound. My first experiment was made by transplanting the *pericranium* (from the os frontis) to the *nose*, to reproduce the ossa nasalia, which had been completely destroyed by disease. Notwithstanding it was evident to me that this experiment, carried on as circumstances required it to be, would be regarded as *unphysiological*, yet that could not deter me from making it, since no unfortunate result could occur to the patient. I can form no opinion as to the termination, yet I do not hesitate to communicate at this time the principal features of the operation, at the same time, however, calling attention to the unfavorable circumstances under which it was necessarily performed.

Case.—Mrs. L., 40 years of age, was received into the Clinic at the commencement of the present session, (1859.) More than two years ago an ozœna developed itself, which led, on the one hand, to a perforation of the hard palate; and, on the other, to an entire loss of the bones of the nose, conchæ and septum narium. In consequence of this destruction, the bridge of the nose was completely sunken, and the external uninjured soft parts of the nose were drawn back against the nasal processes of the superior maxilla. Notwithstanding the decided denial of the patient that she had had a primary affection, yet the uneven surface of the cranium, and the means employed before her admission into the Clinic, authorized the conclusion that the destruction of the bones was caused by *syphilis.* A purulent secretion from the mucous membrane of the fauces was arrested by four weeks' treatment with the iodide of potassium. I wished to defer the operation until spring, but was obliged to yield to the pressing solicitations of my patient, and the operation was performed Nov. 17, 1859. The soft parts of the nose were divided by a semicircular or U incision, (or rather in the form of a horse-shoe,) extending from the processus nasalis of the superior maxilla over the nasal cartilage, from one ala nasi to the other, opening completely into the nasal cavity—separating the lower from the upper portion. The point of the nose was then drawn downward and forward, in such a manner that the *tip* nearly touched the lip. Into the wound, thus made, a similarly shaped flap from the forehead was transplanted, whose pedicle or nourishing point was near the inner corner of the right eye, the borders of which were united to the edges of the nasal wound by silver sutures. When I formed the flap from the forehead, I cut, not only through the *skin*, but through the PERICRANIUM to the bone, and the whole together was then separated from the os frontis, by means of a raspatory. This operation differed from others which I have performed for repairing defects of the nose, only in the fact that the *periosteum was detached with the skin*, and formed the *base* of the flap which was transplanted into the space where the nasal bones were wanting. Afterwards, the edges of the wound in the forehead were brought together as much as possible by two sutures, the denuded portion of the os frontis covered with lint, the nasal cavity filled with charpie, and the nose covered with a cold-water compress. I will only state concerning the subsequent history of this case, that to-day, (Nov. 22,) five days after the operation, the *turgescence* and *swelling* of the *rose-colored* flap are much more marked than I have observed in my former cases. The edges of the wound are perfectly healed at nearly all points, only here and there a superficial suppuration. The defect in this operation is, according to my idea, that the periosteal surface of the flap, twisted to cover the opening in the nose, will be in constant contact with the current of air from the nostrils, and as a natural result, must suppurate and granulate. Whether the pericranium remains capable of producing bone under these conditions, seems very doubtful. The denuding of the os frontis, and the possible superficial exfoliation of the same, would hardly be thought of any account, if the design of the operation is thereby accomplished. In cases of complete destruction of the nose, the chances of this operation would be far more favorable. We could cut the skin (surrounding the nasal defect) to the bone, loosen it with the periosteum, and twist it over so that the epidermis would be turned towards the nasal cavity, which would leave the periosteal surface looking upward. *Upon this surface*, for a basis, a flap of skin and periosteum could be transplanted from the forehead, and in this manner the *pericranium* of the frontal flap would lie on the *periosteum* of the facial flap; that is, two periosteal surfaces would be together, and thus render the chances for bony deposit much more favorable. I have frequently, when supplying defects in the nose by frontal flaps, used the surrounding skin in the manner just cited, (but without the periosteum,) to form a lining for the other flaps, particularly, as in this manner I prevented the adhering of the nasal surfaces, which so easily occurs in these operations.

(APPENDIX.)

BERLIN, *January* 31*st*, 1860.

Having seen the operations referred to in this paper, and observed the patients, I am able to give testimony in regard to them. The young man from whom the fibrous polypi were removed from the posterior nares, *via* an opening made by the resection of the right nasal bone and the processus nasalis of the right superior maxilla, left the Clinic perfectly healed. No exfoliation occurred, and only for a few days was there a discharge near the lachrymal sac. The bony union was complete and firm, when I last saw it, about a fortnight since. No irregularity of the bone or mucous membrane could be felt in the nares.

In case 1st, under division 4th, (enchondroma of the under surface of the palatum durum,) there was a most favorable result. The gums and involucrum palati duri healed to the bone, and no exfoliation took place. The cure is complete.

The boy from whom was resected the processus alveolaris went directly to the country, and died (as was communicated by letter) one week after the operation, from peritonitis. No post-mortem was made.

In case 1st, division 5th, in which there was a *transplantation* of the *pericranium*, to supply a defect in the nose, a most satisfactory result has followed. There was complete union by first intention. There was no morbid secretion from the under (pericranial) surface of the flap, which was greatly feared would be troublesome; but this surface seemed to take on the character of mucous membrane. The apprehended exfoliation of the os frontis did not take place, free granulations having quickly sprung directly from the bone, so that now only a small spot remains uncovered by new skin. In fact, Prof. Langenbeck says it has cicatrized as soon as in the cases where the pericranium was left. The nose feels firm, and has a regular form. A small portion of the flap (which was removed in order to raise the nose up a little) was examined under the microscope, and found to contain an abundance of osseous and cartilaginous cells. Several other osteo-plastic operations have been performed since the above paper was written, the history of which I will endeavor to communicate at a later period. W. F. H.

